# Combined change of behavioral traits for domestication and gene‐networks in mice selectively bred for active tameness

**DOI:** 10.1111/gbb.12721

**Published:** 2021-01-07

**Authors:** Yuki Matsumoto, Hiromichi Nagayama, Hirofumi Nakaoka, Atsushi Toyoda, Tatsuhiko Goto, Tsuyoshi Koide

**Affiliations:** ^1^ Mouse Genomics Resource Laboratory National Institute of Genetics Mishima Shizuoka Japan; ^2^ Department of Genetics SOKENDAI Mishima Shizuoka Japan; ^3^ Anicom Specialty Medical Institute Inc., Chojamachi, Yokohamashi‐Nakaku Kanagawaken Japan; ^4^ Division of Human Genetics, Department of Integrated Genetics National Institute of Genetics Mishima Shizuoka Japan; ^5^ Present address: Department of Cancer Genome Research Sasaki Institute, Sasaki Foundation Chiyoda‐ku Tokyo Japan; ^6^ Comparative Genomics Laboratory National Institute of Genetics Mishima Shizuoka Japan; ^7^ Research Center for Global Agromedicine Obihiro University of Agriculture and Veterinary Medicine Obihiro Hokkaido Japan

**Keywords:** active tameness, behavior, domestication, heterogeneous stock, mouse, selective breeding, sexual difference, tameness, transcriptome, wildness

## Abstract

Tameness is a major element of animal domestication and involves two components: motivation to approach humans (active tameness) and reluctance to avoid humans (passive tameness). To understand the behavioral and genetic mechanisms of active tameness in mice, we had previously conducted selective breeding for long durations of contact and heading toward human hands in an active tameness test using a wild‐derived heterogeneous stock. Although the study showed a significant increase in contacting and heading with the 12th generation of breeding, the effect on other behavioral indices related to tameness and change of gene expression levels underlying selective breeding was unclear. Here, we analyzed nine tameness‐related traits at a later stage of selective breeding and analyzed how gene expression levels were changed by the selective breeding. We found that five traits, including contacting and heading, showed behavioral change in the selective groups comparing to the control through the generations. Furthermore, we conducted cluster analyses to evaluate the relationships among the nine traits and found that contacting and heading combined in an independent cluster in the selected groups, but not in the control groups. RNA‐Seq of hippocampal tissue revealed differential expression of 136 genes between the selection and control groups, while the pathway analysis identified the networks associated with these genes. These results suggest that active tameness was hidden in the control groups but became apparent in the selected populations by selective breeding, potentially driven by changes in gene expression networks.

## Graphical abstract

Tameness can be divided into two components: active tameness and passive tameness. We conducted selective breeding for higher scores of active tameness using a wild‐derived heterogeneous stock. In the analyses of nine behavioral traits related to tameness, we found that five traits showed changes in the selective groups compared to the control groups through the generations. We conducted cluster analyses to evaluate the relationship among the nine traits and RNA‐Seq analysis to characterize the molecular network related to tameness. The results suggested that active tameness was hidden in the control groups but became apparent in the selected populations by selective breeding, potentially driven by changes in gene expression networks.

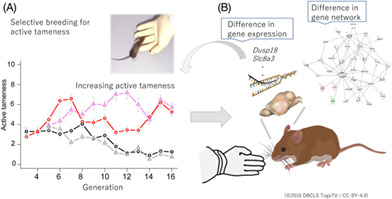

## 1 INTRODUCTION

Tameness is one of the major elements of animal domestication^1^ with a critical role in its early stages.^2^ Tameness has been evaluated using behavioral tests and genetic and/or gene expression analysis in several animal species, such as foxes (*Vulpes vulpes*),^3^, ^4^ rats (*Rattus norvegicus*),^5^, ^6^, ^7^, ^8^ mice (*Mus musculus*),^9^, ^10^, ^11^ and red junglefowl (*Gallus gallus*).^12^, ^13^ These studies demonstrated that genetic factors influence tameness in animals.

Tameness may be divided into two components: the motivation to approach humans (active tameness) and the reluctance to avoid them (passive tameness).^9^, ^14^ In mice, active and passive tameness have been evaluated by using three behavioral tests (active tameness, passive tameness, and stay‐on‐hand tests).^9^ Using these tests, we previously measured nine indices: heading, contacting, locomotion, and jumping in the active tameness test; heading, accepting, locomotion, and jumping in the passive tameness test; and staying time in the stay‐on‐hand test. A difference between wild and laboratory mouse strains was observed in passive tameness but not in active tameness.^9^ These results implied that selective pressure for passive tameness, but not for active tameness, has occurred during mouse domestication. To entirely understand the mechanism of tameness, we need to analyze active tameness in mice.

Based on these results, we previously bred a novel wild‐derived heterogeneous stock (WHS)^15^ that was established by crossing eight wild strains, followed by selective breeding for active tameness.^10^ At the 12th generation of breeding, we obtained selected groups that exhibited a higher level of active tameness than that of the non‐selected groups. Using the selected and non‐selected groups, we conducted genetic analysis and found two genetic loci on chromosome 11 that are associated with active tameness in the selected group. However, it is not clear how the behavioral indices of active and passive tameness relate to each other in terms of behavioral and genetic aspects. In the previous study using data obtained from inbred strains, a relatively higher correlation coefficient was observed between contacting (an index quantifying active tameness) and accepting and staying (indexes quantifying passive tameness), where Pearson's correlation coefficients were 0.55 and 0.60, respectively, but did not achieve significance with the Bonferroni correction.^9^ The study evaluated the correlation between the traits, but more comprehensive analyses were needed to clarify the relationships among traits related to tameness. The link between the brain and domestication also poses a major question. The size of hippocampal regions in domesticated animals, such as sheep and pigs, is smaller compared with those in their wild species counterparts.^16^, ^17^ In foxes, animals selected for higher tameness showed up‐regulation of adult neurogenesis in the hippocampus.^17^ A 2‐fold increase in gene expression for epigenetic regulation has also been reported in domesticated foxes compared with non‐selected foxes.^18^ The hippocampus is a brain region known to be affected by domestication and stress in several species, such as rats and foxes,^17^, ^19^ and varied gene expression levels were observed after stress in mice^20^ and chicken.^21^ Therefore, it is of interest to compare hippocampal gene expression levels and behavior in selected and non‐selected animals.

In the course of selective breeding, the three tests (active tameness, passive tameness, and stay‐on‐hand) were conducted in each generation. Our previous paper reported on the selective breeding,^10^ where we demonstrated heading and contacting in active tameness between generation 3 (G3) and G12, as selective breeding was conducted for these two indices; the first index was the contacting and the second was the heading. Given that further generations of selective breeding up to G16 increased the difference between selected and non‐selected groups, conducting comprehensive analyses of behavioral indices for tameness as well as gene expression in the hippocampus would be valuable.

In this study we first analyzed the sex and generation effect for each behavioral index, then conducted analysis of the change of behavioral indices as well as cluster analyses based on correlation between all tameness‐related indices. Finally, we performed RNA‐seq analysis from mice brains to uncover the differences in gene expression and to identify the associated gene networks between the mouse populations that potentially affect tameness.

## 2 MATERIALS AND METHODS

### 2.1 Rearing condition and animal experiments

Mice were maintained in accordance with guidelines of the National Institute of Genetics (NIG) in Japan. All procedures were carried out with permission from the Committee for Animal Care and Use of the NIG (No. 26–9). All mice were bred and kept under specific‐pathogen‐free conditions at the NIG with food and water available ad libitum. The mice were kept in a temperature‐controlled room (23 ± 2°C) under 12/12 hours light/dark cycle. All mice were weaned from their parents at 3 weeks of age and housed in same‐sex groups with littermates in standard‐size plastic cages containing wood chips until the tameness tests. When there was only one same‐sex littermate, the mouse was individually housed after weaning. During cage exchange and behavioral tests, each mouse was gently caught by its tail using large tweezers covered with silicon tubing to reduce pain.

### 2.2 Behavioral assay

The active tameness, passive tameness, and stay‐on‐hand tests were conducted to quantify nine indices (active tameness test: heading, locomotion, contacting, jumping; passive tameness test: heading, locomotion, accepting, jumping; stay‐on hand test: staying).^9^, ^11^ The three tests were conducted during the light period using a test apparatus made from gray colored polyvinylchloride of 40 × 40 × 40 cm (O'Hara & Co. Ltd., Tokyo, Japan). The field was illuminated with 100 lux at the center, and video recorded using a digital camera, CX5 (Ricoh Company, Ltd., Tokyo, Japan). Each mouse was tested at 6 weeks of age, with the exception of some animals in generations 14–15, which were tested when they were up to 10 weeks old. The ages of all the animals tested for tameness are indicated in Table S1. An active tameness test was established to measure an animal's active responses to a human's hand. After placing the mouse in the center of the test field, an experimenter then put their hand at the bottom of the field, with their fingers slightly moving, and measured the duration of heading, locomotion, contacting, and jumping for 1 minute. The passive tameness test was established to measure an animal's passive responses to a human's hand and was conducted immediately after the active tameness test. An experimenter put their hand at the bottom of the test field without moving the fingers during the 1 minute trial. The hand then chased the mouse slowly until touching the body of the mouse. If the mouse accepted being touched, the experimenter attempted to touch the mouse for as long as possible during the 1 minute trial. The durations of heading, accepting, and jumping were counted. The stay‐on‐hand test quantified behavioral responses to forced stimulation by a human hand and was conducted immediately after the passive tameness test. An experimenter picked up the mouse by the tail with large tweezers and placed the mouse on their hand; while the mouse stayed on the hand, the experiment's thumb stroked the back of the mouse softly at a frequency of once per second. The maximum time for staying was 10 seconds in each trial. If the mouse left the hand, the trial was stopped and repeated three times. The staying times were counted for each of three trials and their median was used as the staying time data. The number of mice used in this study are summarized in Table 1.

**TABLE 1:** Number of animals used in the analysis of the behavioral indices related to tameness in mice

Genaration	Sex	Number of animals			
		C1	C2	S1	S2
3	Female	53	N/A	80	N/A
	Male	50	N/A	80	N/A
4	Female	44	N/A	68	N/A
	Male	42	N/A	66	N/A
5	Female	55	40	53	42
	Male	51	45	57	45
6	Female	67	74	69	65
	Male	69	62	72	58
7	Female	61	62	63	53
	Male	56	67	67	57
8	Female	73	69	73	51
	Male	70	67	77	58
9	Female	59	70	64	61
	Male	54	76	69	55
10	Female	73	64	66	72
	Male	70	72	69	65
11	Female	66	62	67	63
	Male	64	61	69	63
12	Female	71	71	71	71
	Male	66	72	67	64
13	Female	62	73	73	59
	Male	66	65	73	59
14	Female	43	38	57	61
	Male	37	36	53	56
15	Female	39	34	52	47
	Male	34	39	56	47
16	Female	43	48	72	70
	Male	38	48	73	45

Behavioral assays in the different generations (generations 3‐7, generations 8‐12, and generations 13‐16) were conducted by three independent experimenters who were trained to perform similar hand movements. To do this, a video observer checked the procedure of the tameness test until the experimenters showed similar movements. Video observations were conducted by a single, third party individual.

### 2.3 Wild‐derived heterogeneous stocks

**FIGURE 1:**
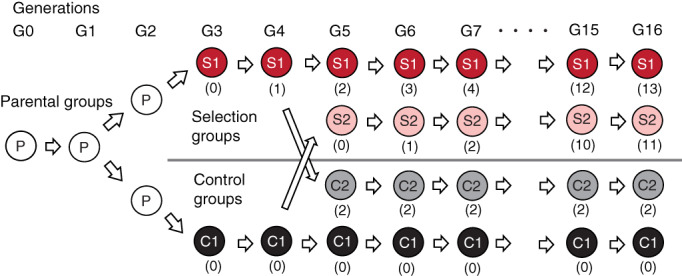
The process of composing two selected groups, S1 and S2, and two control groups, C1 and C2. Numbers in parentheses indicate the number of selections in each group in each generation. Red and pink boxes indicate selection groups, while black and gray indicate control groups

WHS of mice were previously established^10^ and derived from eight wild mouse strains: BFM/2Ms, PGN2/Ms, HMI/Ms, BLG2/Ms, NJL/Ms, KJR/Ms, CHD/Ms, and MSM/Ms. As a result of large genetic differences among these strains, the WHS has higher genetic diversity compared with other existing heterogeneous stocks of mice. Details on the genetic characteristics of WHS are reported in a previous publication.^10^ The eight wild strains were genetically mixed by crosses using the circular rotation rule followed by the random rotation rule from G3 to avoid intercross mating (Figure 1). At G2, pairs in a group were expanded from 8 to 16 and the group was also expanded into two groups, each of which consisted of 16 pairs. At the G3 generation, the genomes of all eight strains were mixed randomly in each mouse, so genetic diversity was expanded more extensively than in the eight founder strains. After G3, 16 pairs of crosses were made by randomly choosing the pairs from the 16 families to avoid intercrossing in each generation.

### 2.4 Selective breeding

Using the WHS, selected groups and control groups without any selection were established and bred as described in the previous study.^10^ Briefly, the selective breeding experiment was conducted using WHS mice to increase active tameness. The mice that exhibited the highest score for contacting in the active tameness test among five mice were used for breeding. In the case where the scores for contacting were identical in the two mice with the highest score, the mouse that showed a higher score for heading in the active tameness test was used for the breeding. These mice were then mated between different families.

We expanded the WHS stock into two groups, selected group named Ms:WHS‐S1 (S1) and control group named Ms:WHS‐C1 (C1) at G3. These two groups, S1 and C1, were each expanded into a further two groups, control group named Ms:WHS‐C2 (C2) and selected group named Ms:WHS‐S2 (S2), respectively, from G5 (Figure 1). For the WHS, up to 80 mice of each sex were tested from each group (C1, C2, S1, and S2) at each generation.

In this study, we used the data for two behavioral indices (heading and contacting in the active tameness test, from G3 to G12), which was published in the previous study.^10^ Data from the other seven indices for G3 to G12 and all nine indices for the following four generations (G12 to G16) are unique to this study.

### 2.5 Evaluation for the effect of sex

The effect of sex in the nine tameness‐related behavioral indices was evaluated using two‐way analysis of variance (ANOVA). The effects of sex, generation, and/or sex × generation interaction were assessed in four groups of WHS mice. Multiple comparisons were corrected using the Bonferroni correction (nine behavioral traits, *P* < .006). We used the *lm* and ANOVA functions implemented in R version 3.3.2 for the analyses.

### 2.6 Evaluation of group differences

The variation of each trait through the generations was tested using the parametric linear regression test (*lm* function in R version 3.3.2) with Bonferroni correction for multiple comparison (0.05/12 [generations] * 6 [group pairs] * 9 [traits]). We applied the test to four groups of WHS (C1, C2, S1, and S2) for each generation from G5. In this analysis, we combined data from male and female mice.

### 2.7 Clustering analysis

The four groups of WHS mice were separated and the individual scores analyzed. In total, data collected from 437 mice at G16 were used for the analysis. The distance between clusters was calculated using the Ward method as implemented by the *hclust* function in R version 3.3.2. The significance was set at 5% for each clustering analysis using *pvclust* function in the R package *pvclust* v2.2.0.

### 2.8 RNA‐seq experiments

Ten mice from each of the four WHS groups at G16–G17 were evaluated via RNA‐seq experiments. Mice were chosen from different mating pairs in the 16 breeding pairs in each WHS group. Male mice were used for these experiments to avoid possible effect of estrous cycle on gene expression in the brain. The animals used in the RNA‐seq analysis were assessed by the three tameness tests, euthanized, and dissected within 15 min following the tests, between 14.00 and 17.00. We extracted total RNA from the hippocampus of mice associated with tameness‐related behavior.^22^ Total RNA was extracted using TRIzol Reagent (Thermo Fisher Scientific Inc. MA, USA) based on the product protocol and TURBO DNase (Thermo Fisher Scientific Inc. MA, USA) was used to degrade genomic DNA. Samples with an RNA integrity number exceeding 7.0 were used for subsequent experiments. Using the standard protocol of Illumina TruSeq series, mRNAs were sequenced on the Illumina HiSeq 2500 platform, generating 100 bp paired‐end reads. Quality control, including removal of adapter sequences, was carried out on the Trim Galore v0.5.0 platform (http://www.bioinformatics.babraham.ac.uk/projects/trim_galore/), using the default settings as of September 22, 2020. We obtained an average of twenty‐six million reads per sample (22 million to 32 million reads per sample). Accession numbers for the data submission are shown in the subsection, data availability statement.

Raw read data (FASTQ files) was mapped to the standard mouse reference genome (GRCm38). Transcripts per million (TPM) values were calculated to measure the gene expression level followed by analysis with a program kallisto, version 0.46.0.^23^ We compared TPM values for two control and select group pairs (C1 vs S2 and C2 vs S1), because the experiments were replicated to allow comparison between groups that were more closely related. In addition, TPM values for a pair of combined control groups and selected groups were compared. The R software (version 3.6.1) and sleuth package (version 0.30.0)^24^ were used for differential gene expression analysis. Gene annotation information was added using the Ensembl genome browser, version 101. The log2 fold changes (beta values), *P*‐values, and q‐values of gene expression for each test were computed based on the Wald test implemented in sleuth and this analysis' significance was set as q‐value <0.05. The differentially expressed genes (DEGs) thus obtained and all genes from each comparison pair were clustered using the *heatmap* function in R software.

Ingenuity pathway analysis (IPA; version 57662101, Ingenuity Systems, Redwood City, CA, USA) was used for the network analysis on 136 genes in the IPA database that were differently expressed between the selected and control groups, based on q‐value <0.05 computed via sleuth analysis as described above. The dataset includes data on mice nervous system tissues whose confidence levels were “Experimentally Observed” or “predicted with high confidence.” The network analysis was conducted using canonical pathway information.

### 2.9 Functional characterization of differentially expressed genes in the human GWAS catalog database

We used the GWAS catalog database to identify the functions of the differentially expressed genes that were found in both mice and humans.^25^ The GWAS catalog file (v1.0.2 ‐ with added ontology annotations, GWAS Catalog study accession numbers and genotyping technology) was downloaded from https://www.ebi.ac.uk/gwas/docs/file-downloads (as of September 22, 2020). We searched for seven genes that commonly showed different expression levels between two control vs selected pairs (C1 vs S2 and C2 vs S1) using “MAPPED_GENE” as a key in the file. To carry out functional annotation of each gene and obtain datasets with matching genes, we searched the catalog using “MAPPED_TRAIT” as a key. Original R scripts were used to summarize the data.

## 3 RESULTS

### 3.1 Generation and sex effects in tameness‐related indices

**FIGURE 2:**
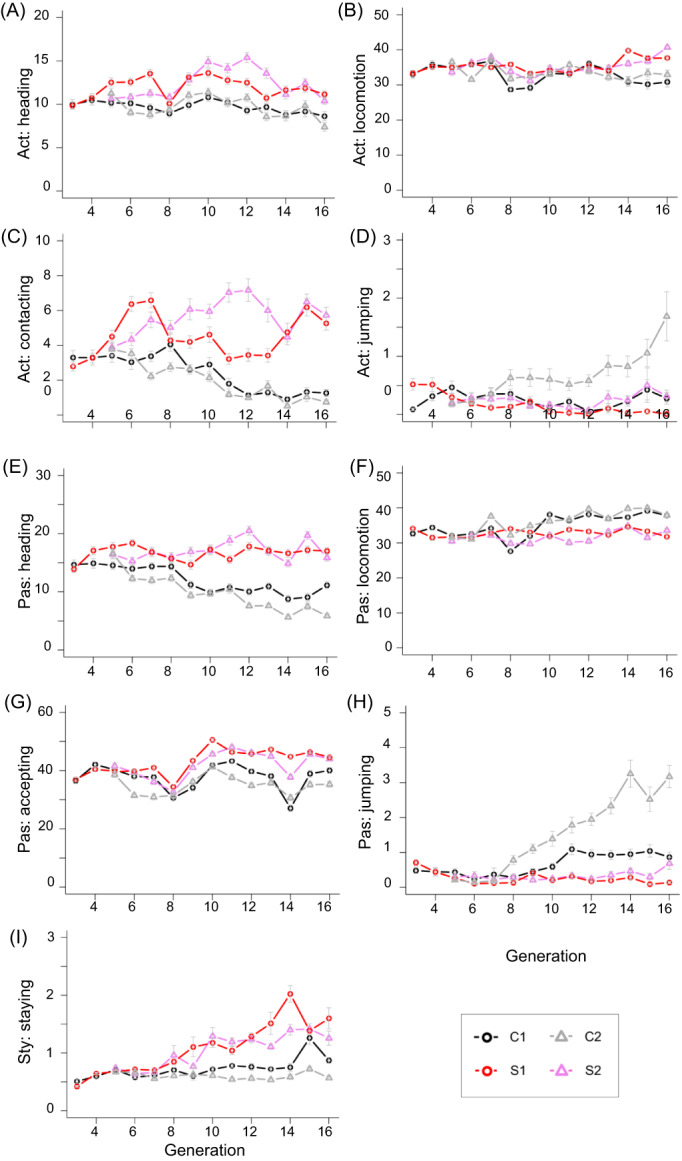
Fluctuation pattern of nine behavioral indices obtained from three tameness tests in WHS mouse over 16 generations. S1 (red) and S2 (pink) are selected groups, while C1 (black) and C2 (gray) are non‐selected (control) groups. Scores show durations (seconds) in A, heading; B, locomotion; C, contacting; and D, jumping in the active tameness test; E, heading; F, locomotion; G, accepting; and H, jumping in the passive tameness test; and I, staying in the stay‐on hand test. Act: active tameness test; Pas: passive tameness test; Sty: stay‐on‐hand test. Error bars indicate mean ± SD

To examine how selective breeding affects the behavioral phenotype in mice, we analyzed changes in behavioral indices related to tameness with increasing generations (Figure S1–S4, Table 1, Table S1). There was no effect of sex in either the control or selection groups, except for locomotion in the active tameness test, accepting in the passive tameness test, and staying in the stay‐on‐hand test in the C1 group, and heading in active tameness test in the C2 group (Tables 2 and 3, Figure S1–S4). A generational effect was detected in five out of nine indices in the S1 group and six indices in the S2 group (Figure 2, Table 3). There were significant generational effects on the six indices in each of the C1 and C2 groups, even though no index was selected. (Figure 2, Table 2). Interestingly, contacting in the active tameness test and heading in the passive tameness test decreased through the generations in the control groups, C1 and C2 (Figure 2) as reported in the previous report.^10^

**TABLE 2:** Results of two‐way ANOVA for nine tame traits in wild‐derived heterogeneous stock control groups (C1 and C2)

Test	Trait	Effect	C1						C2					
			Df	Sum_Sq	Mean_Sq	F_value	Pr_F	Sig	Df	Sum_Sq	Mean_Sq	F_value	Pr_F	Sig
Active tameness test	Heading	Generation	1	157.420	157.420	6.210	.013		1	224.645	224.645	8.447	.004	*
		Sex	1	57.727	57.727	2.277	.131		1	199.135	199.135	7.488	.006	*
		Generation × sex	1	13.644	13.644	0.538	.463		1	8.168	8.168	0.307	.580	
		Residuals	1572	39847.205	25.348				1411	37526.045	26.595			
	Locomotion	Generation	1	1118.463	1118.463	10.065	.002	*	1	348.543	348.543	3.722	.054	
		Sex	1	1325.652	1325.652	11.929	.001	*	1	175.530	175.530	1.874	.171	
		Generation × sex	1	62.837	62.837	0.565	.452		1	44.885	44.885	0.479	.489	
		Residuals	1572	174689.234	111.125				1411	132130.675	93.643			
	Contacting	Generation	1	1149.828	1149.828	88.308	< .001	*	1	1112.110	1112.110	161.570	< .001	*
		Sex	1	6.992	6.992	0.537	.464		1	0.369	0.369	0.054	.817	
		Generation × sex	1	0.268	0.268	0.021	.886		1	8.924	8.924	1.297	.255	
		Residuals	1572	20468.415	13.021				1411	9712.153	6.883			
	Jumping	Generation	1	1.174	1.174	1.667	.197		1	132.664	132.664	39.121	< .001	*
		Sex	1	1.784	1.784	2.534	.112		1	7.404	7.404	2.183	.140	
		Generation × sex	1	0.012	0.012	0.017	.896		1	2.947	2.947	0.869	.351	
		Residuals	1572	1107.030	0.704				1411	4784.922	3.391			
Passive tameness test	Heading	Generation	1	5305.661	5305.661	114.297	<.001	*	1	9207.300	9207.300	251.466	< .001	*
		Sex	1	24.117	24.117	0.520	.471		1	8.325	8.325	0.227	.634	
		Generation × sex	1	208.023	208.023	4.481	.034		1	76.557	76.557	2.091	.148	
		Residuals	1572	72972.023	46.420				1411	51662.992	36.614			
	Locomotion	Generation	1	7481.713	7481.713	156.098	< .001	*	1	7371.494	7371.494	169.133	< .001	*
		Sex	1	301.224	301.224	6.285	.012		1	0.116	0.116	0.003	.959	
		Generation × sex	1	57.329	57.329	1.196	.274		1	11.550	11.550	0.265	.607	
		Residuals	1572	75345.176	47.930				1411	61496.908	43.584			
	Accepting	Generation	1	25.557	25.557	0.225	.635		1	361.610	361.610	3.174	.075	
		Sex	1	968.752	968.752	8.525	.004	*	1	287.742	287.742	2.525	.112	
		Generation × sex	1	132.077	132.077	1.162	.281		1	256.071	256.071	2.247	.134	
		Residuals	1572	178633.666	113.635				1411	160773.567	113.943			
	Jumping	Generation	1	85.489	85.489	51.187	< .001	*	1	1346.407	1346.407	296.731	< .001	*
		Sex	1	0.322	0.322	0.193	.661		1	1.048	1.048	0.231	.631	
		Generation × sex	1	0.196	0.196	0.117	.732		1	0.492	0.492	0.109	.742	
		Residuals	1572	2625.432	1.670				1411	6402.357	4.537			
Stay‐on‐hand test	Staying	Generation	1	18.977	18.977	77.487	< .001	*	1	0.423	0.423	1.734	.188	
		Sex	1	1.873	1.873	7.647	.006	*	1	0.004	0.004	0.015	.901	
		Generation × sex	1	0.152	0.152	0.619	.432		1	0.077	0.077	0.314	.575	
		Residuals	1572	385.002	0.245				1411	344.466	0.244			

Abbreviations: Df, degrees of freedom; Sum_Sq, sum of squares; Mean_Sq, mean squares; Pr_*F*, *P*‐value of *F* statistic; Sig, significance.

**TABLE 3:** Results of two‐way ANOVA for nine tame traits in wild‐derived heterogeneous stock selection groups (S1 and S2)

Test	Trait	Effect	S1						S2					
			Df	Sum_Sq	Mean_Sq	*F*_value	Pr_*F*	Sig	Df	Sum_Sq	Mean_Sq	*F*_value	Pr_*F*	Sig
Active tameness test	Heading	Generation	1	43.902	43.902	1.502	.221		1	166.904	166.904	4.715	.030	
		Sex	1	12.556	12.556	0.429	.512		1	151.534	151.534	4.281	.039	
		Generation × sex	1	15.282	15.282	0.523	.470		1	3.377	3.377	0.095	.757	
		Residuals	1873	54759.093	29.236				1382	48923.916	35.401			
	Locomotion	Generation	1	1503.401	1503.401	19.112	<.001	*	1	1208.452	1208.452	18.483	< .001	*
		Sex	1	174.145	174.145	2.214	.137		1	72.314	72.314	1.106	.293	
		Generation × sex	1	39.526	39.526	0.502	.479		1	0.055	0.055	0.001	.977	
		Residuals	1873	147336.267	78.663				1382	90357.013	65.381			
	Contacting	Generation	1	105.890	105.890	4.869	.027		1	295.883	295.883	9.716	.002	*
		Sex	1	30.710	30.710	1.412	.235		1	2.864	2.864	0.094	.759	
		Generation × sex	1	66.017	66.017	3.036	.082		1	1.417	1.417	0.047	.829	
		Residuals	1873	40734.150	21.748				1382	42086.750	30.454			
	Jumping	Generation	1	38.173	38.173	74.060	<.001	*	1	1.094	1.094	0.945	.331	
		Sex	1	1.227	1.227	2.380	.123		1	2.643	2.643	2.282	.131	
		Generation × sex	1	0.052	0.052	0.101	.750		1	2.380	2.380	2.055	.152	
		Residuals	1873	965.400	0.515				1382	1600.514	1.158			
Passive tameness test	Heading	Generation	1	208.230	208.230	3.586	.058		1	236.403	236.403	3.732	.054	
		Sex	1	26.983	26.983	0.465	.496		1	1.561	1.561	0.025	.875	
		Generation × sex	1	36.907	36.907	0.636	.425		1	17.705	17.705	0.280	.597	
		Residuals	1873	108763.811	58.069				1382	87536.435	63.340			
	Locomotion	Generation	1	21.729	21.729	0.405	.524		1	763.827	763.827	14.439	< .001	*
		Sex	1	1.742	1.742	0.033	.857		1	13.336	13.336	0.252	.616	
		Generation × sex	1	21.964	21.964	0.410	.522		1	1.722	1.722	0.033	.857	
		Residuals	1873	100398.612	53.603				1382	73108.935	52.901			
	Accepting	Generation	1	16637.834	16637.834	212.644	<.001	*	1	5872.056	5872.056	67.400	< .001	*
		Sex	1	5.784	5.784	0.074	.786		1	228.531	228.531	2.623	.106	
		Generation × sex	1	78.573	78.573	1.004	.316		1	74.359	74.359	0.853	.356	
		Residuals	1873	146548.639	78.243				1382	120403.667	87.123			
	Jumping	Generation	1	16.907	16.907	33.515	<.001	*	1	7.992	7.992	8.208	.004	*
		Sex	1	0.134	0.134	0.266	.606		1	2.737	2.737	2.811	.094	
		Generation × sex	1	1.001	1.001	1.985	.159		1	2.884	2.884	2.962	.085	
		Residuals	1873	944.859	0.504				1382	1345.630	0.974			
Stay‐on‐hand test	Staying	Generation	1	291.166	291.166	200.825	<.001	*	1	74.988	74.988	76.940	< .001	*
		Sex	1	0.944	0.944	0.651	.420		1	7.007	7.007	7.190	.007	
		Generation × sex	1	0.336	0.336	0.232	.630		1	0.522	0.522	0.536	.464	
		Residuals	1873	2715.558	1.450				1382	1346.925	0.975			

Abbreviations: Df, degrees of freedom; Sum_Sq, sum of squares; Mean_Sq, mean squares; Pr_*F*, *P*‐value of *F* statistic; Sig, significance.

### 3.2 Behavioral differences between selected and control groups

**FIGURE 3:**
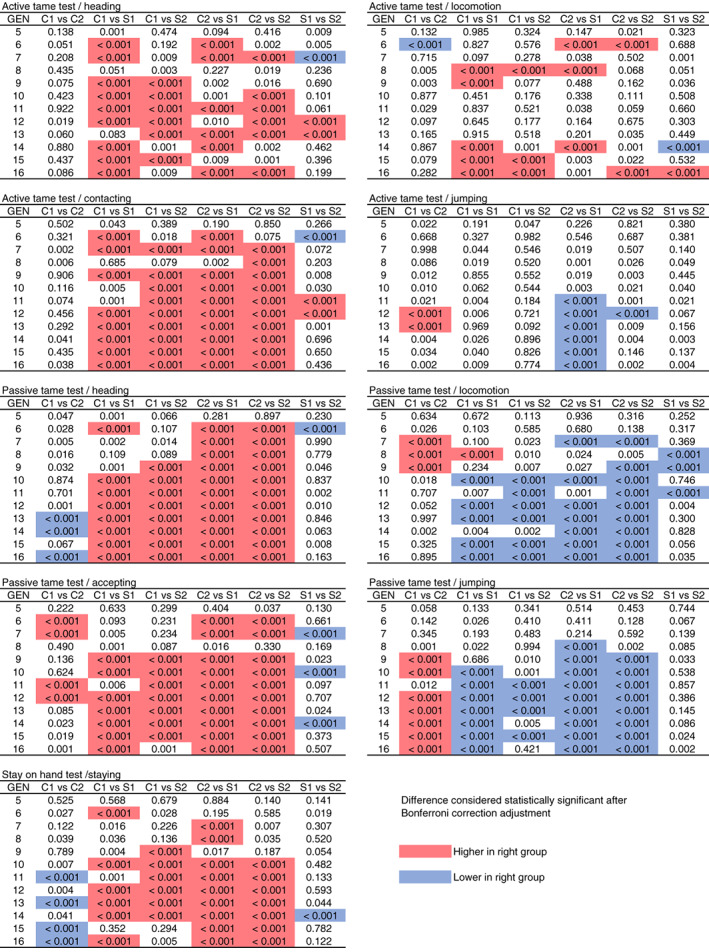
Phenotypic differences in nine behavioral indices between two groups. Differences in the durations of nine indices between all combinations of groups were evaluated over 16 generations. Blue cells indicate lower values in the group on the right side of the comparison. Red cells indicate higher values in the group on the right side of the comparison

Behavioral differences between selected and control groups were evaluated over the generations (Figures 2 and 3, Table 1). Given that the control groups were bred without any selection for tameness but showed changes of tameness‐related indices as the generations proceeded, the difference between the selected and control groups may show a true effect of selection for active tameness (Figure 3). For the majority of the five indices (heading and contacting in the active tameness test, heading and accepting in the passive tameness test, and staying in the stay‐on hand test), durations in the control groups were lower than those in the selection groups after G9 (Figure 3). However, the control groups were higher than the selection groups in two indices (locomotion and jumping) after G10 in the passive tameness test (Figure 3).

### 3.3 Identification of components of behavior in tameness tests

**FIGURE 4:**
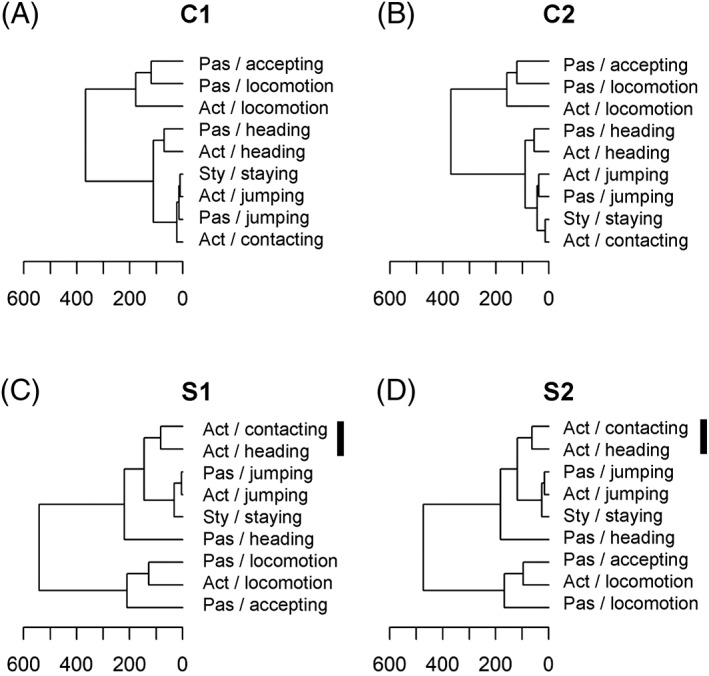
Clustering analyses for nine behavioral indices in four groups of wild‐derived heterogeneous stocks (WHS). Dendrograms in two control groups C1, A and C2, B and two selected groups S1, C and S2, D. The horizontal axis indicates distance as calculated by the Ward method. The vertical bars indicate significant clusters for contacting and heading in the active tameness test (*P* < .05). Act: active tameness test; Pas: passive tameness test; Sty: stay‐on‐hand test

We hypothesized that the phenotypic association between each index related to tameness is high when a significant cluster is observed among the indices. A behavioral/genetic base for the behavioral components may then be shared among the indices. To identify common behavioral factors associated with tameness, we conducted clustering analyses of nine behavioral indices for the two selected groups and two control groups. Two large clusters were found in the WHS control groups, C1 and C2 (Figure 4). The first cluster includes accepting and locomotion in the passive tameness test and locomotion in the active tameness test and the second cluster contains other indices. In the two selection groups, S1 and S2, in addition to the first cluster still present, another cluster including two major selection indices, contacting and heading in the active tameness test, was prominent (*P* < .05). The difference in the clusters between the control and selection groups may be because of more contacting and heading behavior in the active tameness test in the selected groups, so these two behaviors are more notable in the selected groups than in the control groups.

### 3.4 Gene expression profiles

**FIGURE 5:**
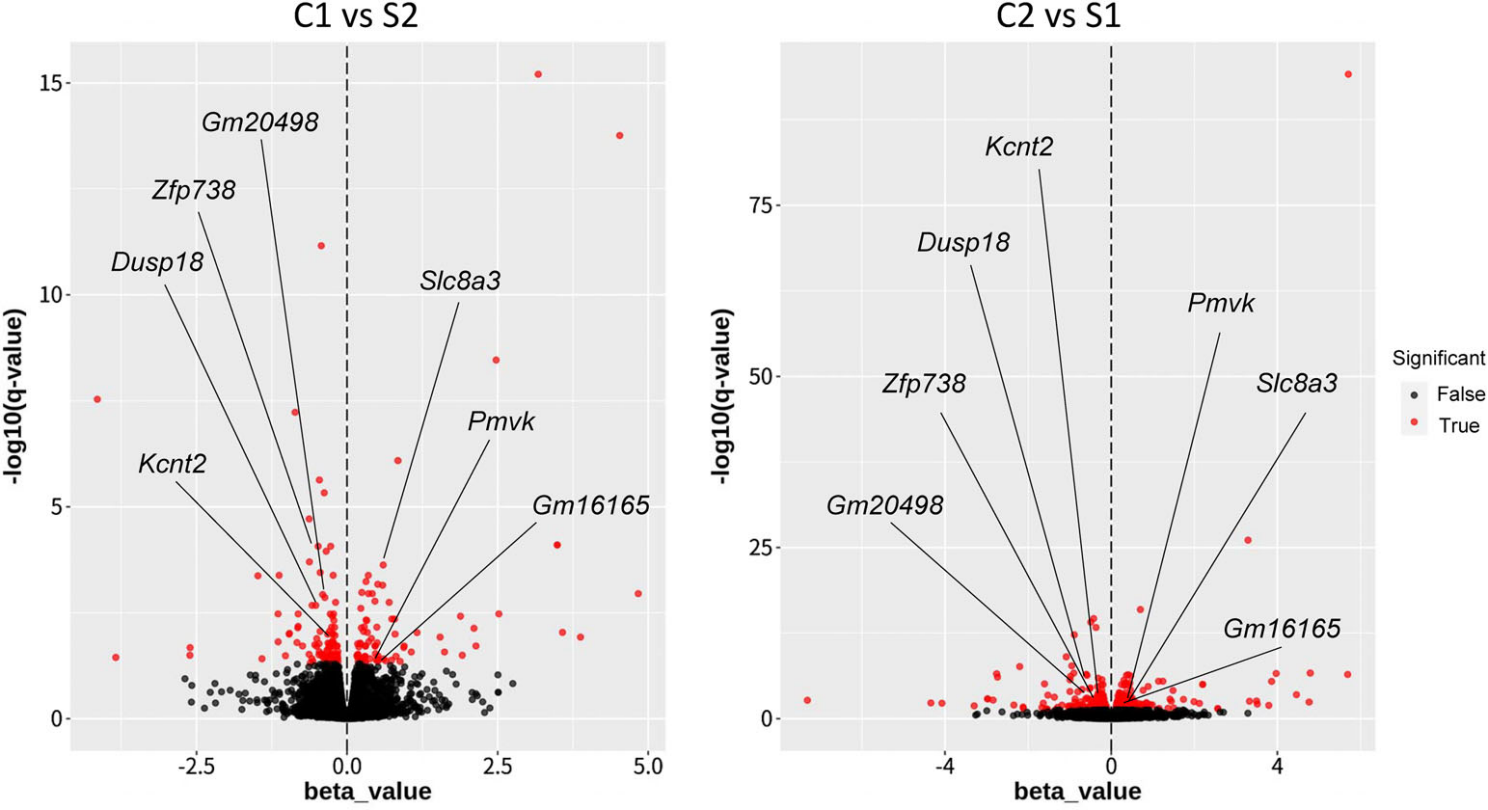
Volcano plots showing differentially expressed genes between control and selected groups, C1 vs S2 using 16,038 genes, A and C2 vs S1 using 16,322 genes, B. Red indicates differentially expressed genes (q values <0.05) in the selected groups compared with control groups

**FIGURE 6:**
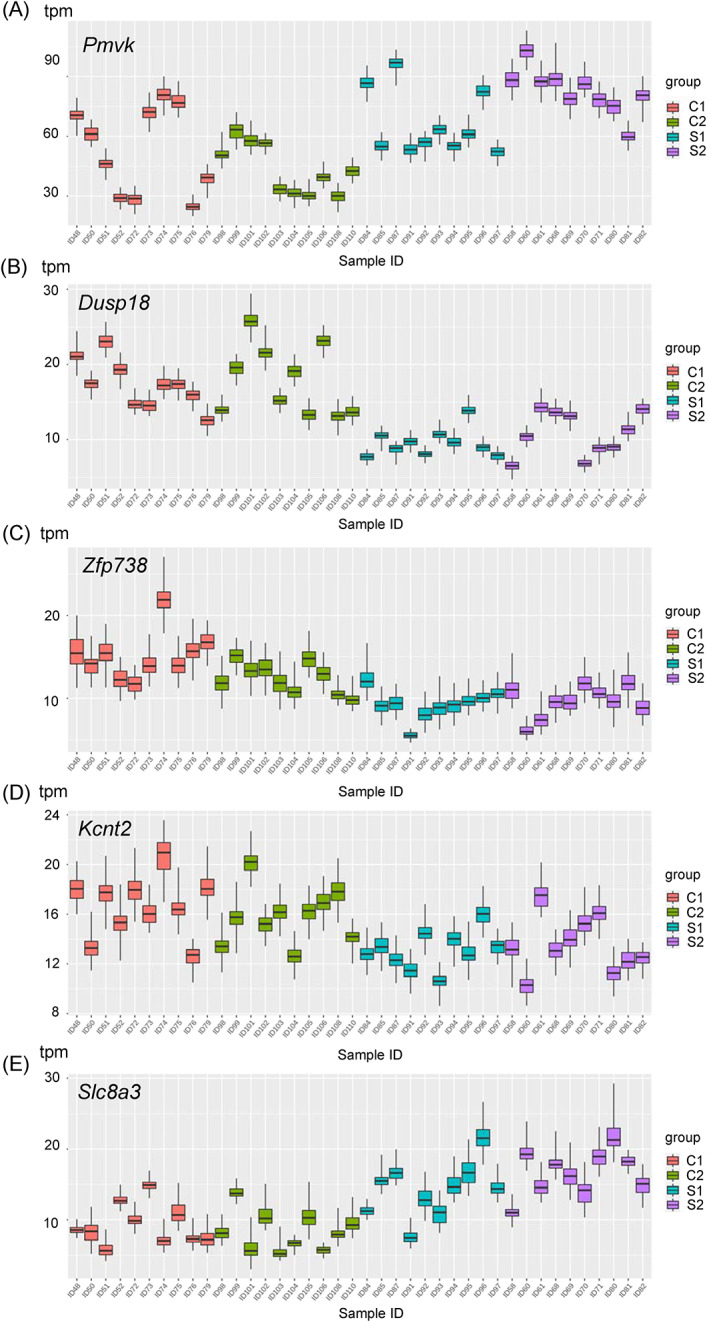
Expression levels of genes that are differentially expressed between control and selected groups in each sample. Boxplots illustrate transcripts per million (TPM) and bootstrap values for five differentially expressed annotated genes: A, *Pmvk*; B *Dusp18*; C, *Zfp738*; D, *Kcnt2*; and E, *Slc8a3* in each sample

To analyze gene expression levels in the hippocampus under selective breeding, we conducted RNA‐seq analysis using samples obtained from 10 animals from each group of C1, C2, S1, and S2. The heatmap of all the 28,675 genes analyzed is shown in Figure S5. No obvious difference in expression levels between the selection and control groups were observed in the heatmap. We found differential expression in 136 genes among the 15,982 genes assessed in C1, C2, S1, and S2 groups (Table S2, Figure S6). Because S1 was expanded from C2 and S2 from C1 (Figure 1), C1 is more genetically similar to S2 than S1, while C2 is more genetically similar to S1 than S2. Therefore, we also compared expression differences in two comparative pairs, C1 vs S2 (Figure 5A) and C2 vs S1 (Figure 5B). We found differential expression in 184 of 16,038 genes assessed in the C1 vs S2 groups (Table S3) and 494 of 16,322 genes assessed in the C2 vs S1 groups (Table S4). Compared with the control groups, 107 (C1 vs S2) and 194 (C2 vs S1) genes had lower expression in the selected groups, although expression of 77 (C1 vs S2) and 300 (C2 vs S1) genes was higher in the selected groups. We found that seven genes, *Gm20498*, *Pmvk*, *Dusp18*, *Zfp738*, *Kcnt2*, *Slc8a3*, and *Gm16165*, exhibited expression change in the same direction between groups in two pairs, C1 vs S2 and C2 vs S1 (Figures 5 and 6, Table 4).

**TABLE 4:** Genes exhibiting expression change in the same direction between C1 vs S2 and C2 vs S1 groups of mice

Higher expression	Gene ID	Gene name	Chr	Start	Stop
Control group	ENSMUSG00000052726	*Kcnt2*	1	140,246,158	140,612,067
Selected group	ENSMUSG00000027952	*Pmvk*	3	89,454,541	89,469,013
Selected group	ENSMUSG00000089958	*Gm16165*	9	22,669,734	22,670,528
Control group	ENSMUSG00000047205	*Dusp18*	11	3,895,240	3,901,296
Selected group	ENSMUSG00000079055	*Slc8a3*	12	81,197,915	81,333,180
Control group	ENSMUSG00000021139	*Gm20498*	12	81,358,860	81,532,905
Control group	ENSMUSG00000048280	*Zfp738*	13	67,658,685	67,687,071

**FIGURE 7:**
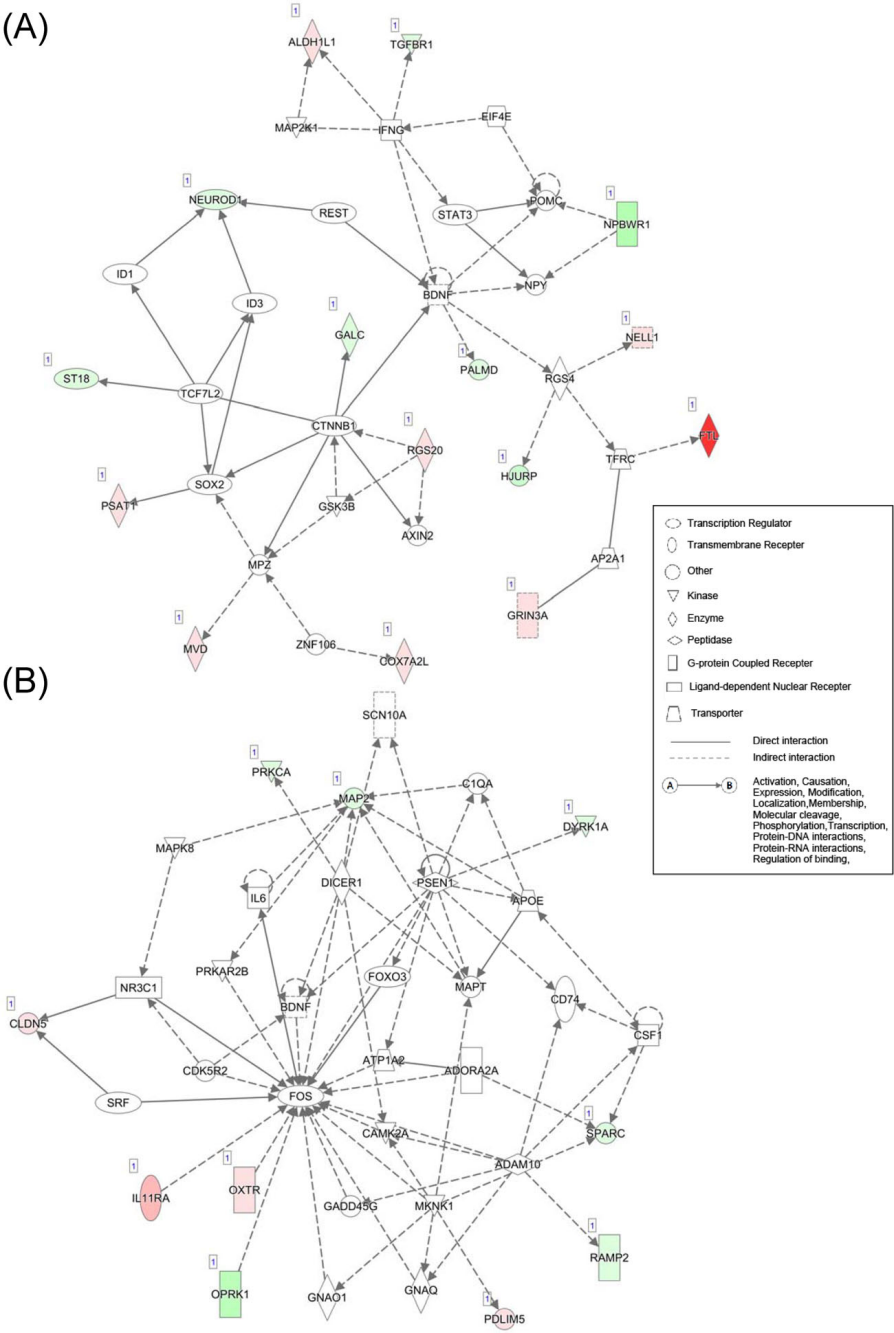
The top two networks of expressed genes identified by IPA analysis. Information on network shapes, path designer shapes, and network relationship are shown in the box. Diagrams shown with green and red colors indicate down‐ and up‐regulated genes with significant differences between groups, respectively. A, ID1 network that scored 13. Thirty‐five genes are involved and associate with behavior, neurological disease, and organismal injury and abnormality. B, ID2 network that scored 11. Thirty‐five genes are involved and associated with cellular development, cellular growth and proliferation, and nervous system development and function

We then conducted IPA analysis to identify the molecular pathways that were different between selected and control groups. The IPA analysis identified 14 networks that are associated with the differentially expressed genes (Table 5). We found 96 genes including canonical pathways; the list of genes associated with the 14 networks are shown in Table S5. Among them, two networks showed relatively high scores, 23 and 13 (Table 5 and Figure 7). The scores indicated the consistency of the upstream regulatory elements, data sets, and their function. The function of the top network with 35 molecules is related to “cellular development, nervous system development and function, and tissue morphology”. The second network with 35 molecules is related to “behavior, neurological disease, organismal injury and abnormalities”. Interestingly, both networks share the brain derived neurotrophic factor (BDNF), which plays an important role as a molecular component in neurogenesis and the maintenance and synaptic plasticity of neural cells.

**TABLE 5:** Expression networks for differential gene expression

ID	Molecules in network	Score	Focus molecules	Top diseases and functions
1	ALDH1L1(↑), AP2A1, AXIN2, BDNF, COX7A2L(↑), CTNNB1, EIF4E, FTL, GALC(↓), GRIN3A(↑), GSK3B, HJURP(↓), ID1, ID3, IFNG, MAP2K1, MPZ, MVD(↑), NELL1(↑), NEUROD1(↓), NPBWR1(↓), NPY, PALMD(↓), POMC, PSAT1(↑), REST, RGS20(↑), RGS4, SOX2, ST18(↓), STAT3, TCF7L2, TFRC, TGFBR1(↓), ZNF106	23	15	Cellular development, nervous system development and function, tissue morphology
2	ADAM10, ADORA2A, APOE, ATP1A2, BDNF, C1QA, CAMK2A, CD74, CDK5R2, CLDN5(↑), CSF1, DICER1, DYRK1A(↓), FOS, FOXO3, GADD45G, GNAO1, GNAQ, IL11RA, IL6, MAP2(↓), MAPK8, MAPT, MKNK1, NR3C1, OPRK1(↓), OXTR(↑), PDLIM5(↑), PRKAR2B, PRKCA(↓), PSEN1, RAMP2(↓), SCN10A, SPARC(↓), SRF	13	10	Behavior, neurological disease, organismal injury and abnormalities
3	KMT2D, Rsl1(↑) (includes others)	2	1	Cell morphology, cellular development, embryonic development
4	DIO2, KREMEN1(↑)	2	1	Dental disease, dermatological diseases and conditions, developmental disorder
5	EIF4E, TMEM119(↓)	2	1	Cell cycle, cellular movement, dna replication, recombination, and repair
6	CRKL, RAPGEF1(↑)	2	1	Cellular movement, hematological system development and function, immune cell trafficking
7	KLK3, SERPINB6(↑)	2	1	Auditory disease, hereditary disorder, molecular transport
8	KCNA6(↑), SUB1	2	1	Cardiovascular disease, neurological disease, ophthalmic disease
9	GPR37, MAP1LC3A(↑)	2	1	Cell death and survival, cellular development, nervous system development and function
10	mir‐204, TGFB3(↑)	2	1	Cellular movement, connective tissue development and function, developmental disorder
11	GLRA2(↑), NOVA1	2	1	Cell‐to‐cell signaling and interaction, cellular assembly and organization, cellular growth and proliferation
12	ERBB2, MAF, PMVK(↑)	2	1	Cancer, dermatological diseases and conditions, organismal injury and abnormalities
13	CGA, NCOR1(↓), TSHB	2	1	Endocrine system development and function, protein synthesis, small molecule biochemistry
14	ITGA4(↓), ITGB1, ITGB7	2	1	Cellular movement, hematological disease, immunological disease

*Note*: Genes with significantly higher and lower expression in the selection group are indicated by upward and downward arrows, respectively, in parentheses.

### 3.5 Searching GWAS catalog data

Searching through 197,711 records in the GWAS catalog data, we identified 26 records that were associated with the seven candidate genes (Table 6). *SLC8A3* and *PMVK* have been associated with bipolar disorder and Parkinson disease, respectively. Multiple GWAS studies reported that *KCNT2* and *DUSP18* are associated with Alzheimer disease, and *KCNT2* is also associated with chronotype (Table 6). These results indicate the important roles of these genes in brain function.

**TABLE 6:** GWAS catalog data associated with differentially expressed genes in selected and control groups in mice

PUBMED ID	MAPPED_GENE	MAPPED_TRAIT
26691988	KCNT2	Atrophic macular degeneration, age‐related macular degeneration, wet macular degeneration
26634245	KCNT2	Forced expiratory volume, response to bronchodilator
29875488	KCNT2	Blood protein measurement
29875488	KCNT2	Blood protein measurement
30019117	KCNT2	Adolescent idiopathic scoliosis
30804565	KCNT2	Chronotype measurement
30696823	KCNT2	Chronotype measurement
26691988	KCNT2 ‐ CFH	Atrophic macular degeneration, age‐related macular degeneration, wet macular degeneration
23028341	KCNT2 ‐ CFH	Complement C3 measurement
30239722	KCNT2 ‐ CFH	BMI‐adjusted waist‐hip ratio
30575882	KCNT2 ‐ CFH	BMI‐adjusted waist‐hip ratio
30575882	KCNT2 ‐ CFH	Waist‐hip ratio
30239722	KCNT2 ‐ CFH	Waist‐hip ratio
29875488	KCNT2 ‐ CFH	Blood protein measurement
30595370	KCNT2 ‐ CFH	Waist‐hip ratio
32450446	LINC01724 ‐ KCNT2 x AL117329.1	PHF‐tau measurement
32450446	LINC01724 ‐ KCNT2 x AL356022.1	PHF‐tau measurement
31701892	PMVK	Parkinson's disease
32450446	SLC35E4, DUSP18xTEX51 ‐ RNU7‐182P	PHF‐tau measurement
32450446	SLC35E4, DUSP18xTEX51 ‐ RNU7‐182P	Neurofibrillary tangles measurement
23382691	SLC8A3	Serum IgG glycosylation measurement
21602797	SLC8A3	Cystic fibrosis
27989323	SLC8A3	Monokine induced by gamma interferon measurement
22969067	SLC8A3	Corneal topography
31043756	SLC8A3	Bipolar disorder
30595370	SLC8A3	Body height

## 4 DISCUSSION

We conducted selective breeding for active tameness in mice and successfully selected groups that exhibited a high level of active tameness, and thus a high motivation to approach a human's hand. In addition to the active tameness test, we also conducted two more behavioral tests, passive tameness and stay‐on‐hand tests, at each generation for each group. From these behavioral tests, we obtained data for nine behavioral indices. Sexual difference in several mouse behaviors has been reported previously.^26^, ^27^, ^28^ In the present study, however, we found no clear sex effect in nine behavioral indices of tameness in the four WHS groups. Given that mice are externally not highly sexually dimorphic and tameness is not a behavior directly associated with sex, we believe these tameness‐related behaviors did not exhibit a sex difference.

To clarify the relationship among the nine behavioral indices, we conducted clustering analysis and clearly demonstrated that there is a cluster of tameness‐related behaviors in the selected groups of WHS. The cluster of heading and contacting in the active tameness test is closely related with another cluster comprising jumping in active and passive tameness tests and staying in stay‐on‐hand test. In the previous study, we conducted correlation analysis between each pair of indices obtained from tameness tests using wild and laboratory strains^9^ and obtained that seven pairs of indices had significant correlation. Among them, three correlations showed a reasonable match with the current results, where high correlation was observed between heading and contacting in the active tameness test, locomotion in the active and passive tameness tests, and jumping in the active and passive tameness tests. These results suggest that the behavioral indices that have a high level of correlation may be related to each other behaviorally or mechanistically. According to the cluster analysis in WHS, contacting and heading in the active tameness test are closely related.

In addition, the groups that were selected for contacting showed higher heading values compared with control groups (Figures 2 and 3). Furthermore, two other behavioral indices (heading in passive tameness and staying in the stay‐on‐hand test), which are located in the next clusters within the same branch, also showed higher values in the selected groups than the control groups. These results illustrated that contacting and heading in the active tameness test and the other two indices could partially share behavioral and/or genetic components. It is interesting that heading and contacting in the active tameness test are not clearly separated in the control groups but classified into independent clusters in the selected groups, S1 and S2. It is possible that selection for contacting makes the behavioral indices for active tameness more apparent after the selective breeding.

In the previous study, when we established the three behavioral tests, we intended to measure the active and passive tameness separately.^10^ Given that two indices, contacting and heading, in the active tameness test and two indices, accepting and heading, in the passive tameness test, as well as staying in the stay‐on‐hand test, are separated in different clusters, our strategy of using three tameness tests that can measure active and passive tameness separately was validated.

As we previously reported, contacting in the active tameness test decreased in control groups as the generations proceeded.^11^ A similar pattern of decrease in behavioral index in the control groups was observed for heading in the passive tameness test. These results suggested that control groups increased wildness as the generations proceeded. The jumping behavior has been observed frequently in wild strains that have not been selected deliberately but little or not at all in laboratory strains.^9^, ^29^, ^30^, ^31^ Our results suggested that the duration of jumping could be used as an indicator to evaluate mouse wildness. We found lower values in selection groups than in control groups for jumping in the passive tameness test. Decreasing the duration of jumping in both selection groups suggested that the wildness of selected groups should have decreased through the selective breeding. This is supported by the closer relatedness between two clusters, a cluster of contacting and heading in the active tameness test and a cluster of jumping in the active and passive tameness tests, demonstrated in the cluster analysis. In contrast, the duration of jumping increased as the generations proceeded in the control groups where no deliberate selection was applied. A reason for the increase in wildness in non‐selected control groups is not clear. As we speculated in the previous study, random crosses from eight inbred strains decreased the haplotype length thus increasing genetic diversity; therefore, these changes may contribute to increasing wildness.^10^

Several studies have reported gene expression differences in selective breeding for tameness and other phenotype‐based selective breeding experiments. In foxes, tame groups have lower pro‐opiomelanocortin (POMC) expression compared with the non‐selected group.^32^ Another report of RNA‐Seq analysis from rats revealed several candidate genes including *Gltscr2*, *Lgi4*, *Zfp40*, and *Slc17a7* associated with tameness and aggressiveness.^8^ The present study uncovered differential gene expression in 136 genes and in expression networks between populations. We found differential expression in *Dusp18* (Figure 6, Table 4), which affects response to external stimuli. Hyperactivity and abnormal behavioral response to light was observed in mice mutated with a *Dusp18* intragenic deletion (IMPC Database release 2014). For the expression network analysis, we found two gene networks that were clearly different between selected and non‐selected groups. In the first network, we found SOX2 to be the fourth most connected molecule. It has been proposed that domestication of animals is strongly associated with diverse changes in behavioral, morphological, and physiological traits. This is called “domestication syndrome” and the mild deficit of neural crest cells might play a key role in the phenotypic change.^2^ Sox2 is one of the genes having a critical role in the development of neural crest cells during developmental stage.^33^ Although our RNA‐seq analysis targeted gene expression in the hippocampus, it will be valuable to analyze gene expression in neural crest cells in the future. It is interesting that the first gene network consisted of neuropeptides B/W receptor 1, neuropeptide Y, and POMC (Table 5). POMC is expressed as a precursor of the adrenocorticotropic hormone, expressed in response to stress stimuli and also demonstrated to be less expressed in tamed foxes^32^; therefore, a similar mechanism may be expected for tameness in both mice and foxes. In the second network, we identified oxytocin receptor, which plays a key role in social preference and recognition, suggesting an association between social behavior and tameness.^34^, ^35^, ^36^ In addition, FOS, a product of immediate early gene expression activated by stimulus in the neural cells, has the highest number of interactions with other molecules in the second network. Mice selected for higher tameness are thought to be less responsive to external stimuli, e.g., a social encounter with another animal.^37^ Furthermore, although the tissue samples for RNA‐seq analysis were collected within 15 min after the tameness tests (approximately 5 min each), we cannot exclude the hypothesis that the expression of the immediate early gene *Fos* may have started during this time. It is possible that the difference in the effects of neural cell activation, as per the tameness tests, is reflected in the difference between the networks associated with selected and non‐selected groups. Therefore, the role of FOS in the molecular network might be critical to explain the difference in tameness between selected and non‐selected groups. A common gene found in both networks is BDNF, which plays a role in neurogenesis and the maintenance and synaptic plasticity of neural cells (Table 5). BDNF expression is reported to be affected by stress,^38^, ^39^, ^40^ thus it is possible that the difference between tamed and non‐tamed animals is related to stress sensitivity. Thus, further studies are needed to reveal how stress sensitivity affects the difference between tameness and non‐tameness.

We conducted comparative analysis of seven DEGs commonly found in the two sets of RNA‐seq analyses with the results in the previously reported GWAS.^25^ Four of seven differentially expressed genes identified in human GWAS studies, were tentatively reported as loci associated with human neurological diseases: *Slc8a3* for bipolar disorder,^41^ *Pmvk* for Parkinson's disease,^42^ *Kcnt2* and *Dusp18* for Alzheimer's disease,^43^ and *Kcnt2* for insomnia and chronotype traits.^44^, ^45^ These diseases are not directly related to tameness or domestication in animals; however, combined studies on human GWAS and DEGs between tamed and non‐tamed mice indicate that these genes are playing important roles in maintaining a variety of normal functions in the brains.

We previously reported that two closely located genomic regions on chromosome 11, ATR1 and ATR2, were selected in the group of mouse that are selectively bred for tameness using the WHS^10^. We found nine functionally annotated genes, *Wscd1*, *Xaf1*, *Atp2a3*, *Sez6*, *Sdf2*, *Aldoc*, *Vtn*, *Atp5g1*, and *Sp6k*, to be differentially expressed between C1 and S2 groups in the ATR1 and ATR2 regions (Table S3). Also, two genes, *Srr* and *Ramp2*, were differentially expressed between C2 and S1 groups in these regions (Table S4). Thus, it will be important to understand how these genes affect gene expression levels and gene networks in future studies.

In summary, we conducted selective breeding for active tameness and successfully bred tame mice. At the same time, we identified a combined change in other behavioral traits, such as accepting, heading, locomotion, and jumping in passive tameness, and staying in the stay‐on‐hand test. Our cluster analysis demonstrated that the behavioral component of active tameness was notable in the selected groups where contacting and heading in the active tameness test were selected. The results suggested that the behavioral component of active tameness was hidden in other behaviors in the non‐selected group of mice but became apparent in the selected groups. Behavioral components of active tameness may be influenced by other behaviors, such as anxiety, fear, sociability, and novelty‐seeking behaviors.^9^, ^10^ In addition, we found changes in the gene expression pattern between the selected and non‐selected groups. Further analyses will be required to quantify behaviors with the generally used behavioral tests to analyze the relationships between tameness and other behavioral aspects. Furthermore, it is necessary to characterize the neural cells that are activated during contact with human hands to understand the neurobiological mechanism underlying active tameness in mice.

## Supporting information


**Figure S1** Sex differences in nine tame traits in Ms:WHS‐C1.
**Figure S2.** Sex differences in nine tame traits in Ms:WHS‐C2.
**Figure S3.** Sex differences in nine tame traits in Ms:WHS‐S1.
**Figure S4.** Sex differences in nine tame traits in Ms:WHS‐S2.
**Figure S5.** Heatmap of gene expression levels by using all 28,675 genes.
**Figure S6.** Heatmap of 136 genes differentially expressed genes.

**Table S1** Data on nine tameness‐related behavioral indices used in the study.

**Table S2** A list of 136 genes that showed differential expression between the selected and control groups.

**Table S3** A list of 184 genes that showed differential expression between C1 and S2 groups. The genes located in ATR1 and ATR2 genomic regions are highlighted.

**Table S4** A list of 494 genes that showed differential expression between C2 and S1 groups. The genes located in ATR1 and ATR2 genomic regions are highlighted.

**Table S5** A list of genes associated with 14 networks identified in the ingenuity pathway analysis.

## Data Availability

The data that supports the findings of this study are available in the supplementary material of this article and in DDBJ, reference numbers, DRA010533; BioProject: PRJDB9931; BioSample: SAMD00229087‐SAMD00229126; Experiment: DRX228339‐DRX228378; Run: DRR238167‐DRR238206.
