# Supplementary material for: Combined change of behavioral traits for domestication and gene‐networks in mice selectively bred for active tameness
Source: Genes Brain Behav. 2021 Jan 7;20(3):e12721. doi: 10.1111/gbb.12721 (PMC7988575; doi:10.1111/gbb.12721)
Supplement: Supplementary file 1 — Figure S1 Sex differences in nine tame traits in Ms:WHS‐C1. Figure S2. Sex differences in nine tame traits in Ms:WHS‐C2. Figure S3. Sex differences in nine tame traits in Ms:WHS‐S1. Figure S4. Sex differences in nine tame traits in Ms:WHS‐S2. Figure S5. Heatmap of gene expression levels by using all 28,675 genes. Figure S6. Heatmap of 136 genes differentially expressed genes. [file GBB-20-e12721-s005.pdf]

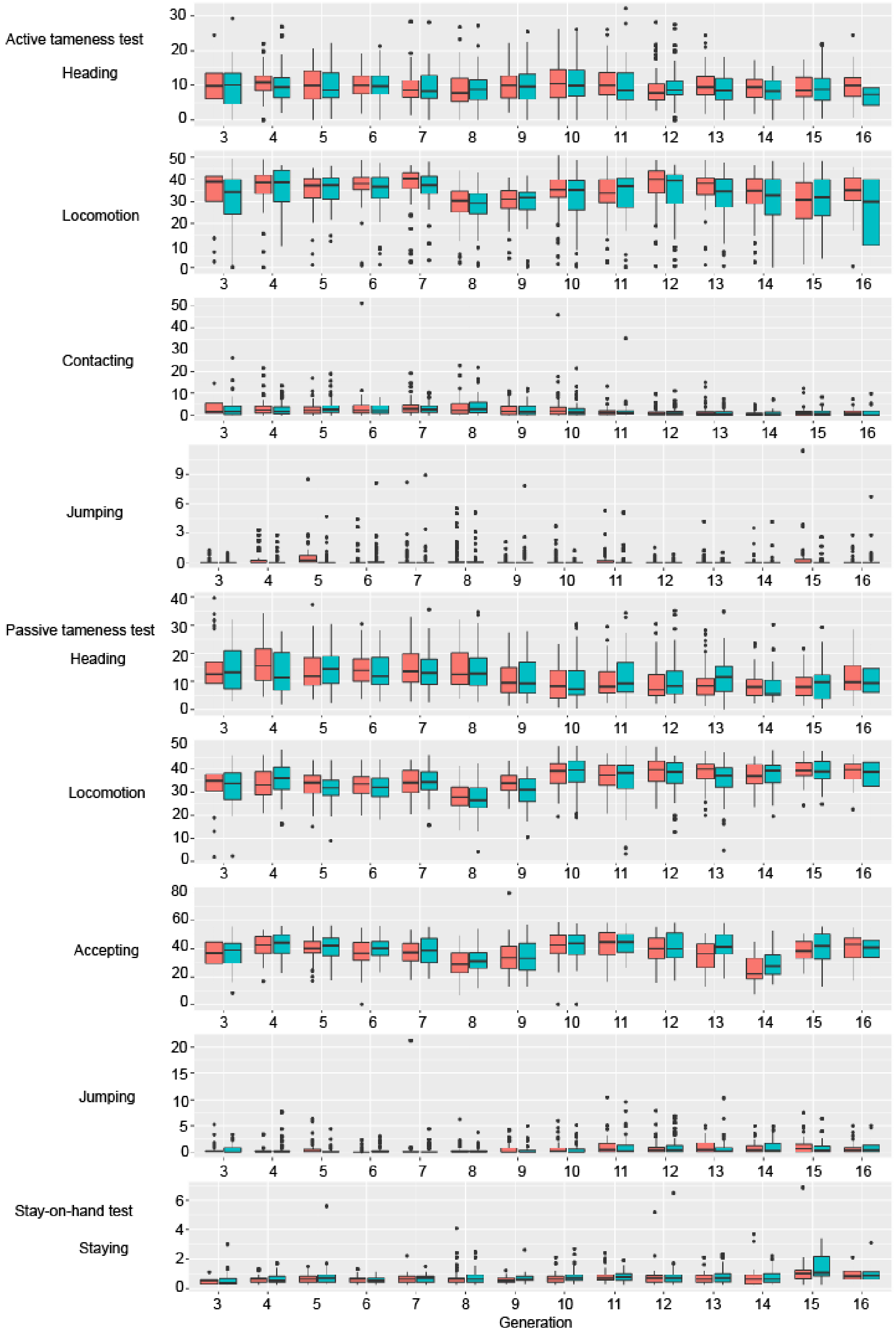

Fig. S1. Sex differences in nine tame traits in Ms:WHS-C1. Females and males are indicated in red and blue bars, respectively. Data are shown in Table S1.

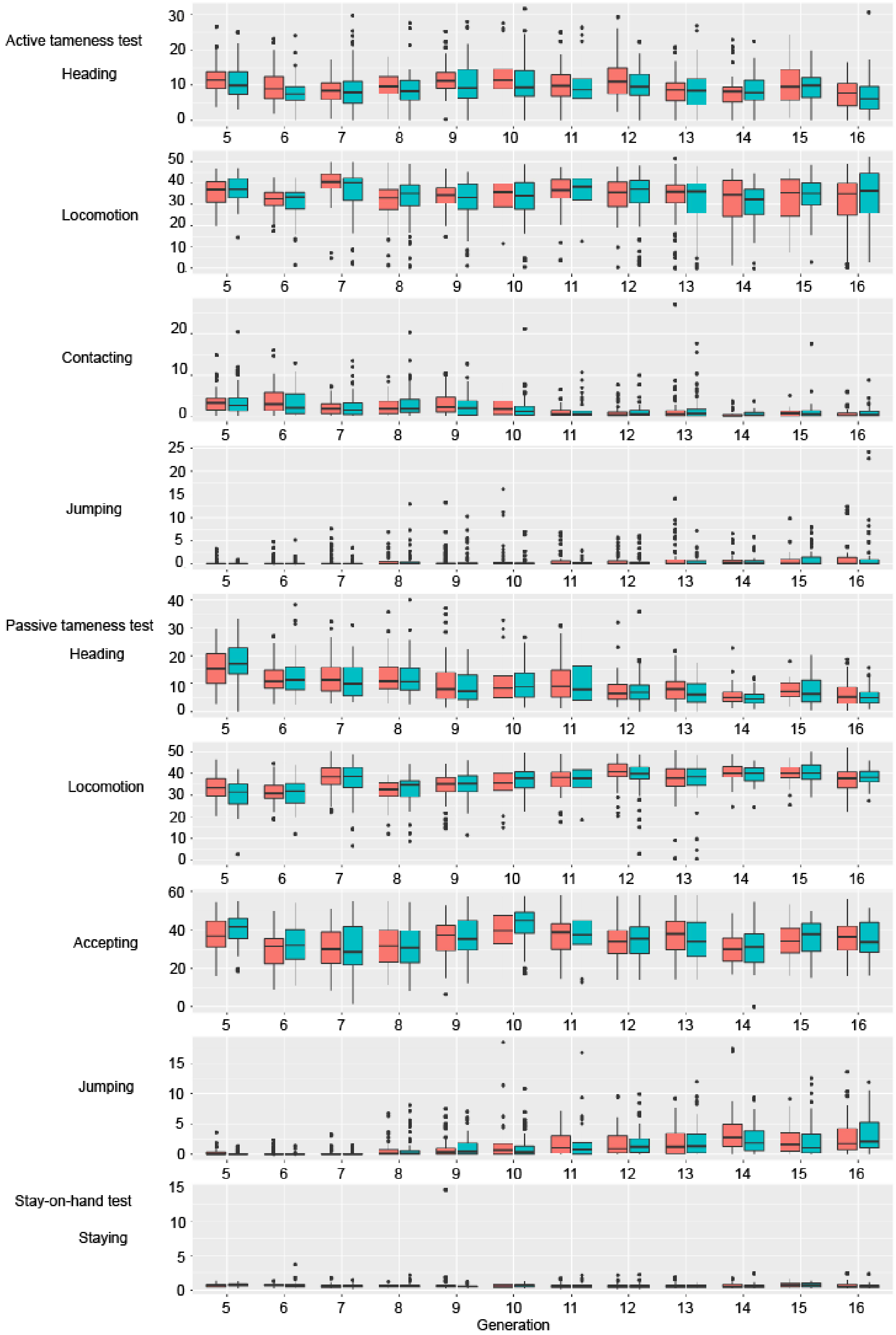

Fig. S2. Sex differences in nine tame traits in Ms:WHS-C2. Females and males are indicated in red and blue bars, respectively. Data are shown in Table S1.

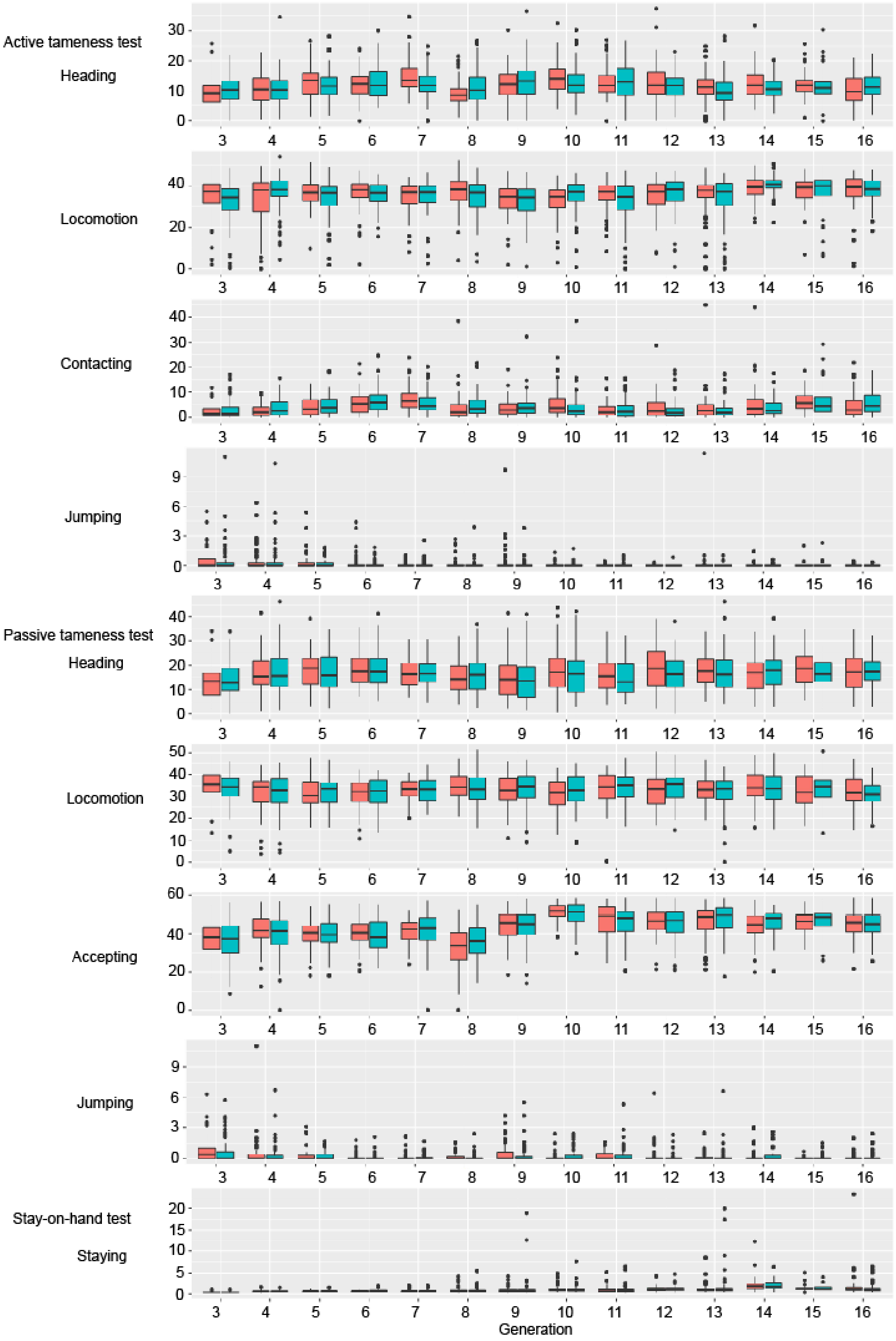

Fig. S3. Sex differences in nine tame traits in Ms:WHS-S1. Females and males are indicated in red and blue bars, respectively. Data are shown in Table S1.

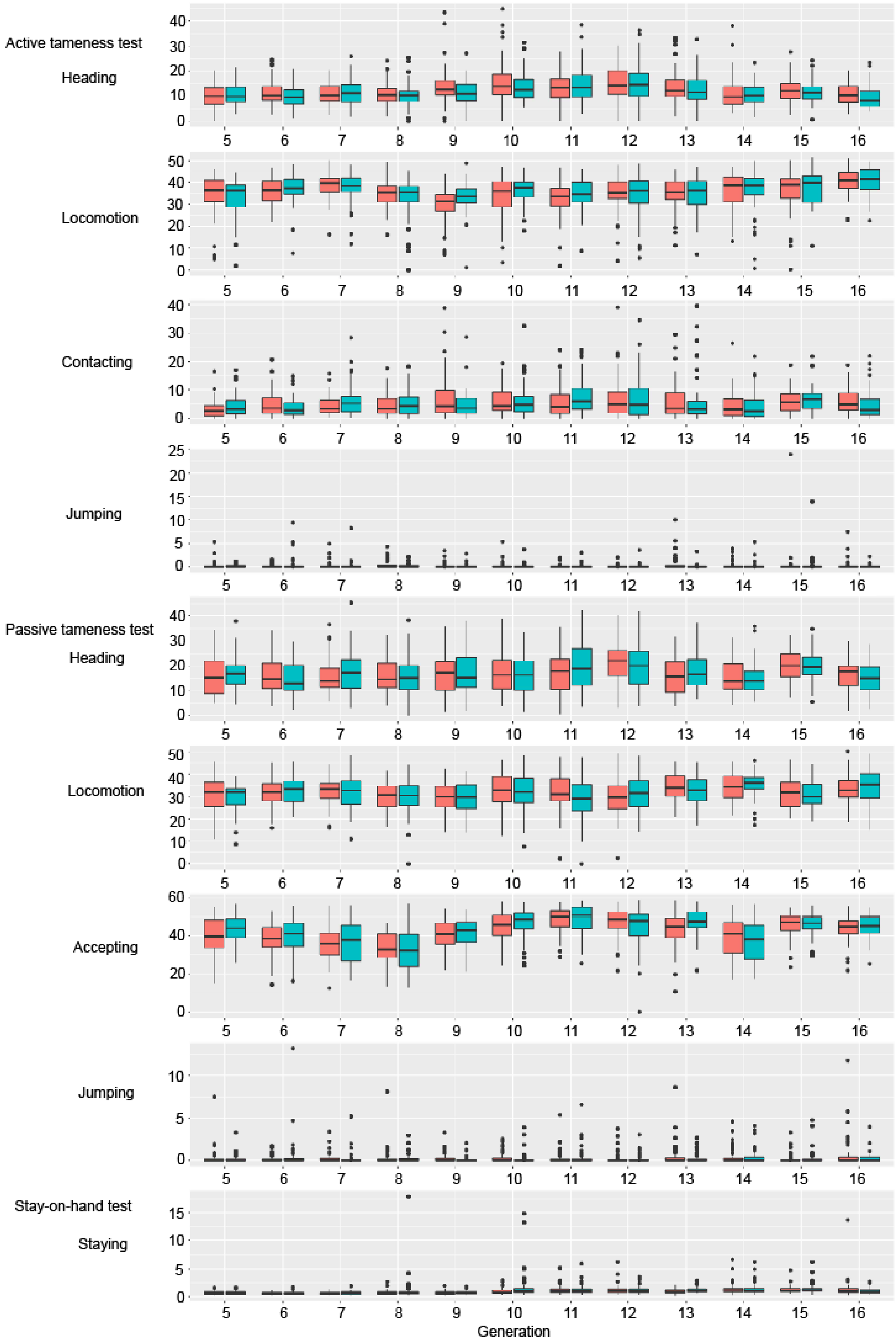

Fig. S4. Sex differences in nine tame traits in Ms:WHS-S2. Females and males are indicated in red and blue bars, respectively. Data are shown in Table S1.

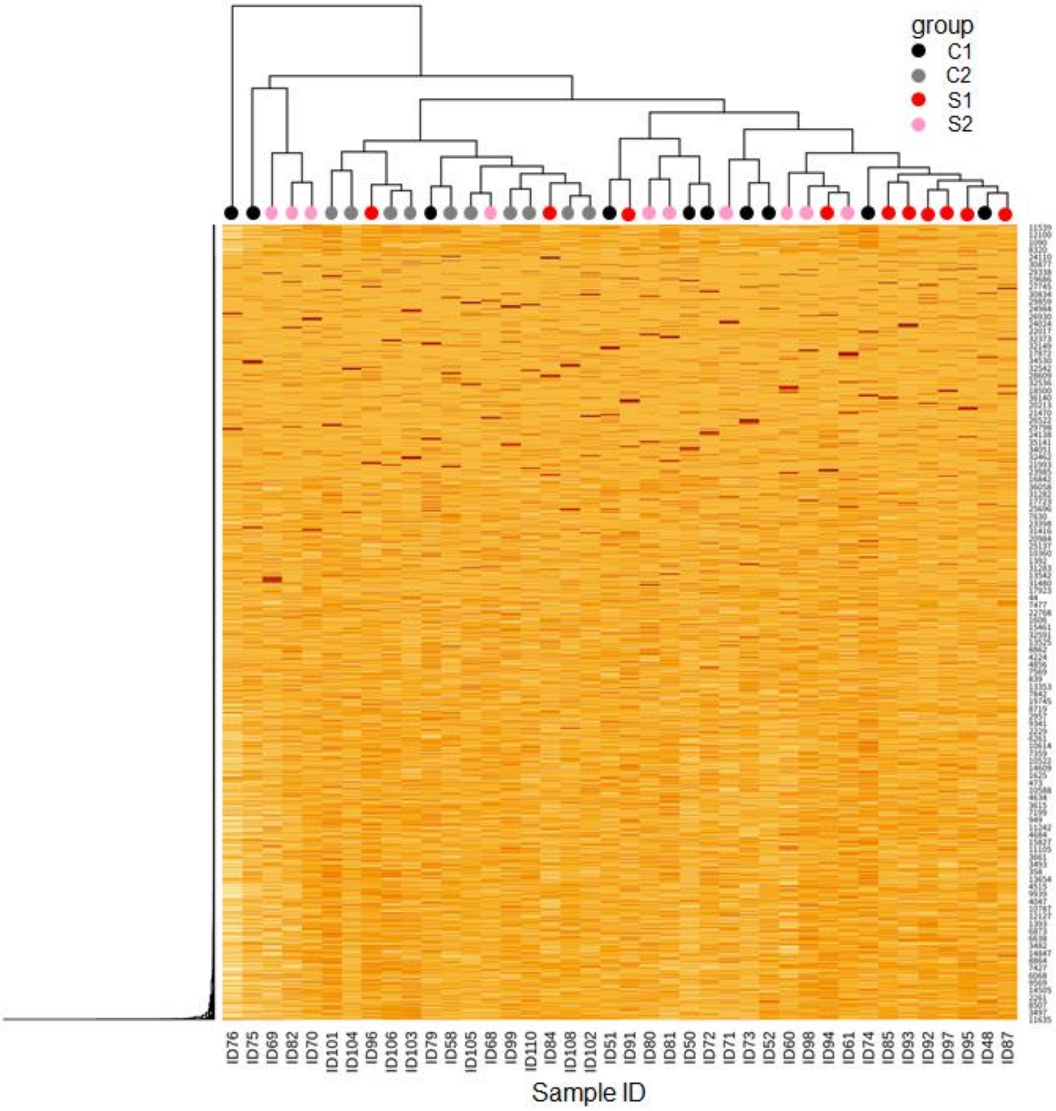

Figure S5. The heatmap of all 28,675 genes. Heatmap showing the clustering pattern of 28,675 genes analyzed for two control groups (C1, C2) and two selected groups (S1, S2) of mice. Each column represents an individual control or selected sample, and each row represents an individual gene.

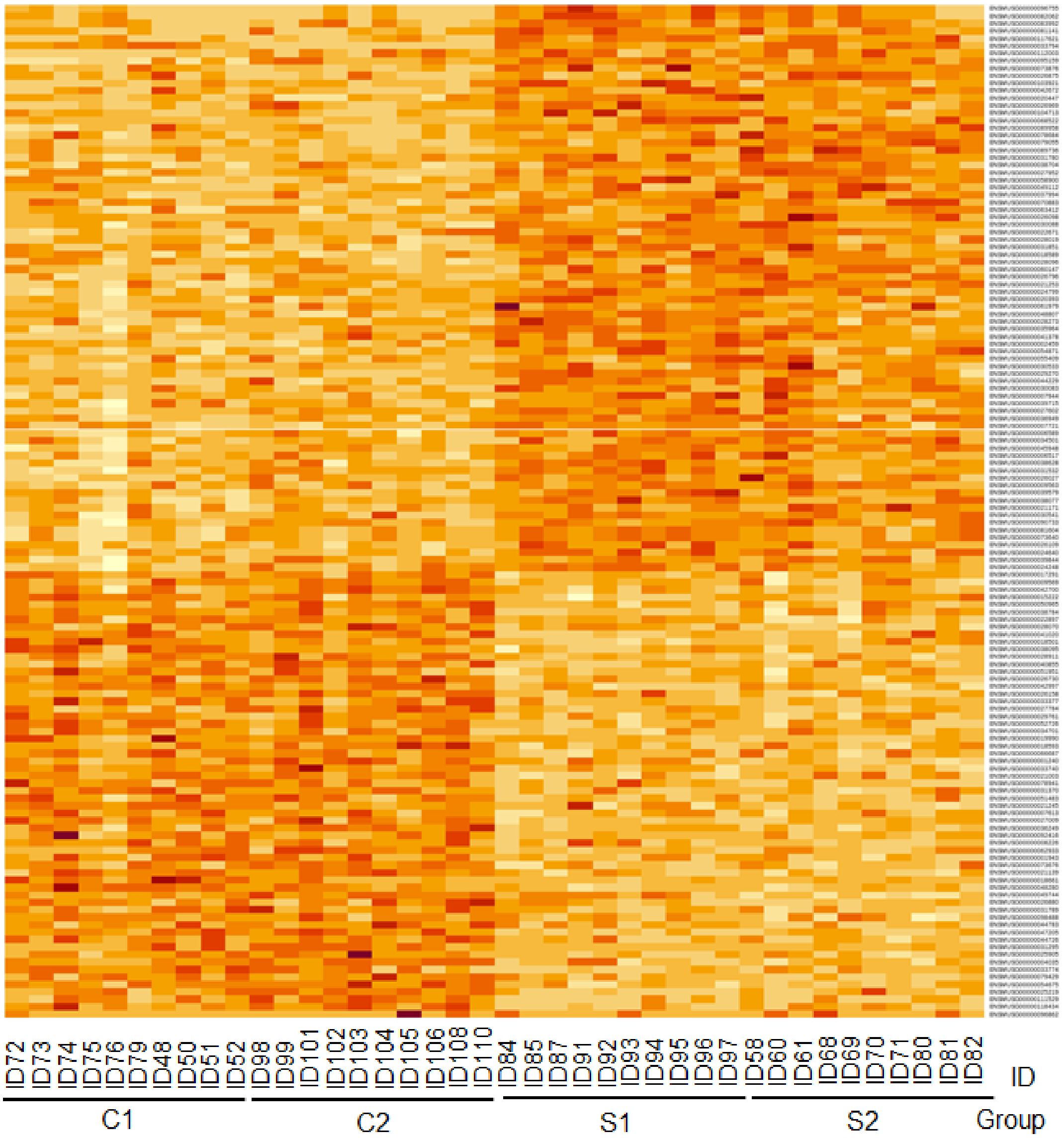

Figure S6. The heatmap of DEGs by using 136 genes. Heatmap showing the expression pattern of 136 genes that were differentially expressed in control (C1, C2) and selected (S1, S2) groups of mice. Each column represents an individual control or selected sample, and each row represents an individual gene. Information on the genes can be found in Table S2.
